# Simulations of a heavy ball falling through a sheared suspension

**DOI:** 10.1007/s10665-017-9935-5

**Published:** 2017-09-15

**Authors:** Adam K. Townsend, Helen J. Wilson

**Affiliations:** Department of Mathematics, University College London, Gower Street, London, WC1E 6BT UK

**Keywords:** Concentrated suspensions, Microstructure, Oscillatory shear, Repulsion, Sedimentation, Simulations, Stokesian Dynamics, Suspensions

## Abstract

In recent experiments, Blanc et al. (J Fluid Mech 746:R4, 2014) dropped a heavy sphere through a concentrated suspension of smaller, neutrally buoyant particles. They found that the application of a lateral oscillatory shear flow caused the heavy ball to fall faster on average, and that for highly concentrated suspensions, at certain moments of the cycle of shear oscillation, the heavy ball moved upwards. We use Stokesian Dynamics to model these experiments and other related scenarios. We show how the motion of the heavy particle and the microstructure of the suspension depend on two key dimensionless parameters: the frequency of the oscillations (relative to a typical settling time) and the strength of repulsive interparticle forces, relative to the buoyancy-adjusted weight of the heavy ball. We offer a mechanism which describes some of the observed behaviours: the formation and breakup of vertical repulsion chains.

## Introduction

Particles sedimenting through viscous media have been the focus of investigations since at least Stokes’ investigation [1] in 1851. Since then, the focus of investigations has moved from a single spherical particle sedimenting in a Newtonian background fluid to multiple, polydisperse particles, sedimenting through a variety of background fluids and in the presence of a variety of background flows.

Questions we can ask about these sedimenting particles include what determines their settling speed, what is the effective viscosity of the suspension through which they fall, and whether any structure forms in the suspension which hinders/aids this sedimentation. For example, Beiser et al. [2] through their experiments found that the settling speed depended on the particle material, chemical forces from the suspending fluid, as well as the suspension concentration. As we could expect, sedimenting particles at low concentrations were observed to fall faster than at high concentrations. However, the dependence on concentration is not monotonic: increasing the concentration led to the formation of particles clusters, which once again increased the sedimentation rate. Increasing the concentration further, however, ultimately slowed these clusters down to below the speed seen for a single particle, given the overwhelming number of interactions.

At small scales, experimental work combines well with simulation work, where experiments provide observations which can then be investigated fully by simulations. In this paper, we perform simulations using Stokesian Dynamics [3], a method well suited to modelling sedimenting particles where inertia can be neglected [4, 5], but modified to allow for spheres of different sizes. Stokesian Dynamics simulations have, in the literature, found good agreement with a number of experiments, going some way to answering some of the questions mentioned above [5]. Stokesian Dynamics has also been used to reproduce results from other numerical methods: Harlen et al. [6] investigated sedimentation through a suspension of neutrally buoyant fibres, validating a dependence for the falling velocity on the fibre concentration.

**Fig. 1:**
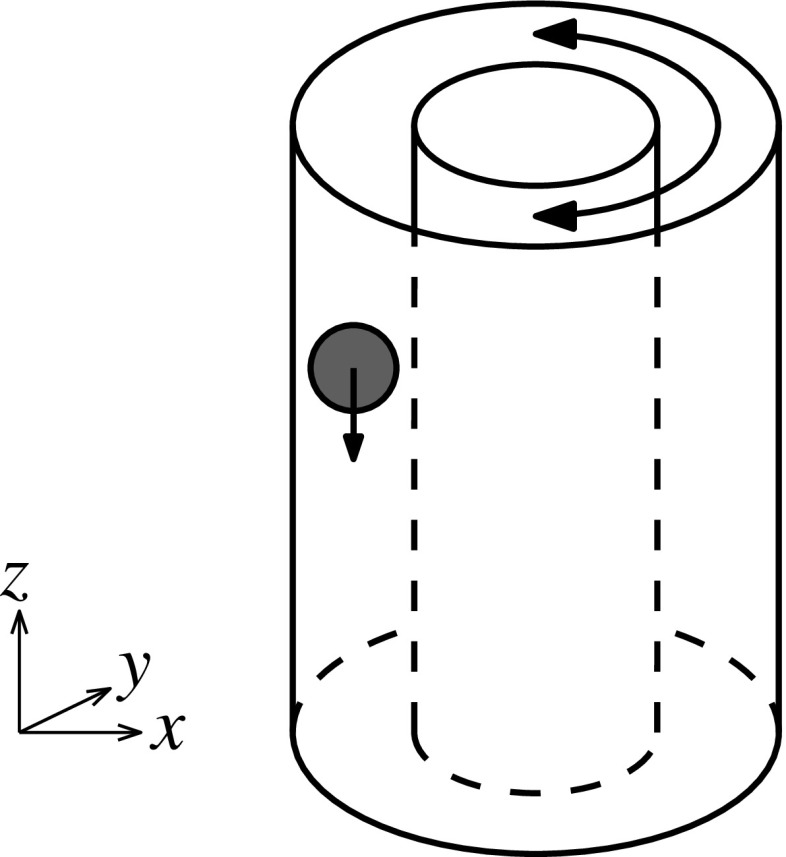
Setup of the Blanc et al. [7] experiment

**Fig. 2:**
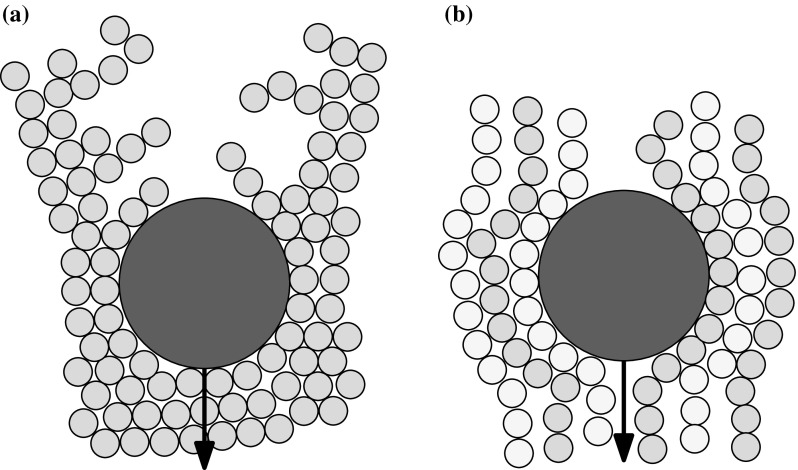
Sketches of the possible microstructure mechanisms involved in the falling-ball experiment. **a** The wake behind the falling particle causes a density disturbance. **b** Induced vertical particle alignment (coloured alternately in *shades of blue*) may make it easier for the ball to travel vertically. (Color figure online)

The particular sedimentation problem which concerns this paper is inspired by the work of Blanc et al. [7]. They recently discovered that a heavy ball falling through a dense suspension of smaller particles can be made to fall at tunable speeds by applying a transverse oscillatory shear to the system, as shown in Fig. 1. The mechanism, they hypothesised, lies in the microstructure created in the small-particle suspension by each flow. A falling ball creates an asymmetric density disturbance, with more particles ahead of it than are found in its wake (Fig. 2a); this naturally hinders its falling. The cross-shear, on the other hand, may encourage the small particles to align in the vertical direction (Fig. 2b), making it easier for the large sphere to pass.

In the first experiment in the paper, the large ball was dropped through a resting suspension with high volume concentrations of $\phi = 0.40$ and 0.47. The mean falling velocity was found to be the same for both a non-presheared suspension and the one treated to a steady preshear. Treating the suspension with an oscillatory preshear was found to make a small difference, increasing the large ball’s fall speed by 5–10%.

**Fig. 3:**
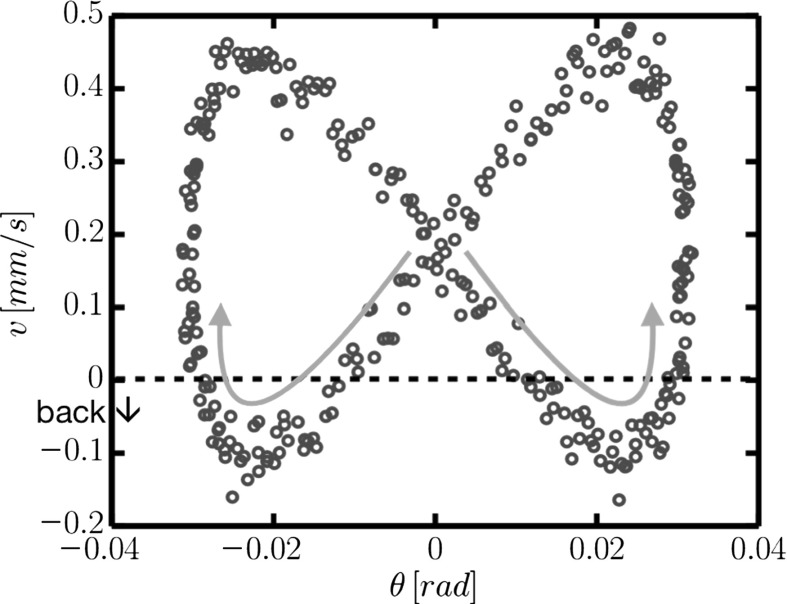
Falling ball velocity over a shear cycle, found by Blanc et al. [8]. Reproduced by permission

In the second experiment, the large ball was dropped through an oscillatory cross-sheared suspension. Tracking the large ball’s fall speed, the mean falling speed was found to increase up to a factor of four, depending on the frequency of the oscillation. They also found [8] that for very concentrated suspensions, when they tracked the large ball’s fall speed during a shear oscillation, its variation was so extreme that at certain moments of the shear cycle, it was actually travelling upwards (see Fig. 3).

Here we aim to reproduce some of these observations numerically, and therefore elucidate the importance of various physical parameters on the two phenomena. Ultimately, we find that we are unable to reproduce the increase of the mean falling speed , although we find suspension structure which agrees with the hypothesis in Blanc et al. [7]; however, we are able to reproduce the large velocity variance over a shear cycle, and give a mechanism for this behaviour.

In Sects. 2–4, we discuss how we set up the simulation to best mimic the experiments, taking into account particle interactions. In Sects. 5–7, we perform the simulations and detail the observed behaviour of the system as we alter parameters in the model. Finally, we offer a mechanism responsible for this behaviour in Sect. 8.

## Simulation setup

We perform simulations using Stokesian Dynamics [3]. We place a large sphere into a box of small, neutrally buoyant particles, where the size ratio is small enough to capture the size difference in the experiments, while not being so small as to require excessively many particles. In all cases, we apply a double-layer electrostatic repulsive force between close pairs of particles to prevent overlap [9]: the role of repulsion forces is discussed in Sect. 3. We use a sufficient number of small particles in the simulations so that the vertical motion at the edges of the box is almost zero: this can be up to 10,000 particles and is discussed in Sect. 4.

We apply shear to the suspension,

1 $$\begin{aligned} {\tilde{\varvec{u}}}^\infty = (\alpha {\tilde{y}} \cos (2 \pi {\tilde{f}} {\tilde{t}}), 0, 0), \end{aligned}$$

in the notation of Blanc et al. [7], through the linear background velocity field2The shear is in the *xy*-plane, with3The motion of the large sphere is then mostly governed by two parameters we will alter in our simulations: the repulsion strength, $\tilde{k}$, and the shear frequency, $\tilde{f}$. We nondimensionalise our lengths on the radius of the large particle, $a_{\mathrm {large}}$, and our forces on the buoyancy-adjusted weight of the large particle, *W*. Times are scaled with the viscous timescale, *T*, associated with the large particle. This is defined such that imposing a unit force on the large particle, in the absence of all other particles, leads to a unit Stokes flow velocity. Therefore, the dimensionless shear frequency, *f*, in terms of the dimensional frequency, $\tilde{f}$, is given by

4 $$\begin{aligned} f = \tilde{f} T = \frac{6\pi \mu a_\mathrm {large}^2 \tilde{f}}{W}. \end{aligned}$$

Thus, if we were to double the weight of the large particle, the effect in our dimensionless system would be to halve both the repulsive force, *k*, and the dimensionless frequency, *f*. The other physical parameters in the experiments are detailed in Table 1, as well as the simulation parameters we use to represent these.

**Table 1:** Physical parameters used in the experiments by Blanc et al. [7], and the values we use in our simulations

		Used	Experiment
Physical parameters			
$\lambda = a_\mathrm {small}/a_\mathrm {large}$	Particle size ratio	1 / 10	1 / 33
$\alpha$	Shear amplitude	1 / 3	Various, greatest effects at $\sim$1/3
$f = \tilde{f} T$	Dimensionless frequency	Free	
*c*	Monolayer 2D area concentration	0.6	
$\phi$	3D volume concentration	0.40	0.40, 0.47
Simulation parameters			
*N*	Number of small particles	$\sim$10,000	Large
$N_p$	Pre-shear oscillations	2	Large
$N_\mathrm {osc}$	Main oscillations	2	Large
$\varDelta t/T$	Timestep	$2\pi / 250f$	Small
$(\tau a_\mathrm {large})^{-1}$	Repulsion decay length	1 / 20	
$k=\tilde{k}/W$	Strength of repulsion force	Free	

**Table 2:**
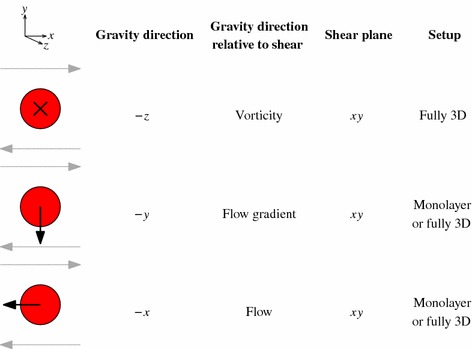
Summary of the three sedimentation and shear directions we simulate. The setup where gravity acts in the negative *z*-direction, with the ball falling into the page, models the Blanc et al. [7] experiment

In the experiment, gravity acts in the vorticity, or negative *z*-direction. Since simulations are such a flexible tool, we have also considered the two other possible orientations of gravity. All three options are illustrated in Table 2. In the two new cases—with gravity in the negative *x*- and *y*-directions—the direction out of the plane of shear is essentially neutral, and we can make a reasonable approximation by considering a single layer—a monolayer—of spheres (in which we parametrise the small-particle suspension in terms of its area concentration rather than the volume fraction). In the monolayer, the particles are naturally confined to the plane as all the forces from the particle interactions act in this plane.

Throughout this paper, we will define the *x*-direction as the flow direction, *y* as flow gradient, and *z* as vorticity for consistency. However, note that these axes will have different orientations relative to gravity in the three different scenarios. We will use words like ‘up’, ‘down’, ‘top’ and ‘bottom’ in the usual layman’s meanings, relative to the direction of gravity in each case.

The experiments in Blanc et al. [7] that we attempt to mimic are set up, with parameters in Table 1, thus:

1. Monodisperse small particles are dispersed in a density-matched Newtonian fluid, which is placed into a Couette cell.
2. The system may be oscillatory presheared (experiments done both with and without).
3. A large heavy sphere is placed in the cell, towards the top.
4. The heavy sphere is dropped along the centreline of the gap, while the cell has oscillatory shear applied to it.
5. The velocity of the falling ball is measured over the following oscillations.

Our simulations, unless otherwise specified, are set up in a similar way:

1. The small particles are randomly distributed in a given box.
2. The large particle is placed inside the suspension, with any overlapping small particles being deleted. No weight force is applied to the large particle yet.
3. The simulation is run for 25 timesteps with no background flow and with only the repulsion force turned on. This allows the particles to equilibrate.
4. The system is presheared for two oscillations, with only the repulsion force turned on. This allows the system to form shear-related structure.
5. Gravity is activated on the large sphere, and it falls through the box for one oscillation, allowing structure to form under and around the ball.
6. With all expected structure formed, the velocity of the ball is now measured over the following oscillation(s).

We are using our own new implementation of Stokesian Dynamics, which has been extensively verified against results from both the original codes of Brady and Bossis [3] and the more recent (and independent) implementation for monodisperse suspensions of Wilson and Davis [10].

## The role of repulsion forces

In simulating concentrated suspensions, we have to be careful of particles overlapping. Lubrication interactions in the Stokesian Dynamics method slow down approaching particles, but without a globally sufficiently small timestep, particles can still sometimes overlap [4, 11]. To counter this, a repulsion model is commonly included. A common choice is a pairwise electrostatic repulsion model, where the force, ${{\varvec{F}}}$, between two spherical particles of radius *a* and *b*, with their surfaces separated by a distance *h* and unit vector displacement $\varvec{d}$, is given by

5 $$\begin{aligned} {\varvec{F}} = \frac{2ab}{(a+b)} \tilde{k}\mathrm {e}^{-\tau h} \varvec{d}, \end{aligned}$$

as in Mari et al. [12]. The strength of repulsion, $\tilde{k}$, is a parameter we can vary. The repulsion decay length, $(\tau a_\mathrm {large})^{-1}$, however, we fix, setting $\tau =20/a_\mathrm {large}$, as in Table 1. Double-layer electrostatic forces are seen in the literature for both non-Brownian and colloidal systems, and are plausible so long as the repulsion decay length is small [12]. Other repulsive force models exist [13]; typically they decay exponentially.

**Fig. 4:**
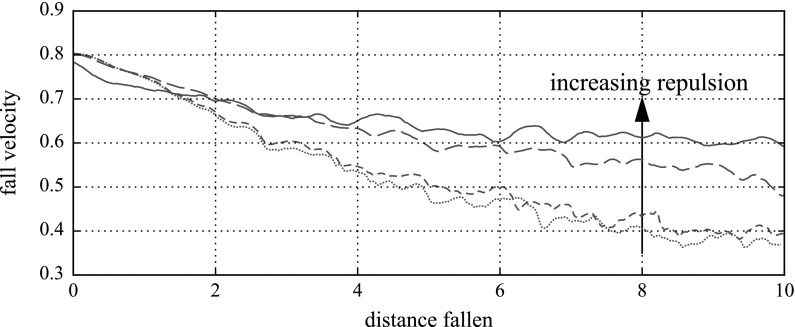
Fall velocity of a large particle sedimenting through an unsheared monolayer suspension of area concentration $c=0.6$, with increasing levels of repulsion. Without repulsion, the suspensions jam quickly, causing the falling sphere to decelerate greatly. The repulsion model used here is Eq. (5) with $\tilde{k} = 0$, 0.02, 0.2 and 2; $\tau = 20/a_\mathrm {large}$, as discussed in the following sections

Without a mild repulsion force, a concentrated system can quickly jam, with the sedimenting sphere unable to pass through. Figure 4 shows the falling speed of a large particle through such an unsheared and initially unrepelled monolayer suspension (i.e. no steps 3 or 4 above), without and with the increasing levels of subsequently applied repulsion. The difference is dramatic: where in the unrepelled system (dotted, bottom line), the falling particle decelerates throughout to less than half of its starting speed as it falls through, and continues to decelerate, in the highest—but still moderate—repelled system (solid, top line), the particle reaches an approximately steady falling velocity of around 75% of its initial speed quite quickly.

**Fig. 5:**
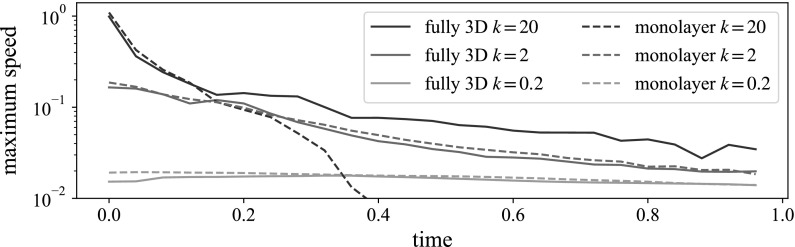
Maximum speed of all particles after enabling repulsion in a randomly distributed cell. After 25 timesteps (corresponding to 1 time unit here), all repulsion strengths lead to, at most, small ‘jiggling’ of particles. Results averaged over two (fully 3D) or three (monolayer) initial random configurations

We apply repulsion to a randomly distributed cell of particles for a time unit (25 timesteps) to allow the particles to equilibrate before we shear the suspension. Figure 5 shows the maximum particle speed over this time frame. Higher repulsive strengths lead to initially larger speeds, but for both the monolayer and fully 3D suspensions, after a time unit, the largest speeds are reduced to a fairly constant ‘jiggling’, which is a property of the timestep.

**Fig. 6:**
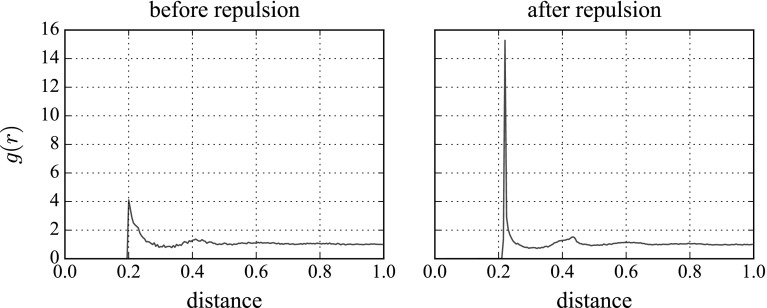
Particle density functions of the small sphere suspension before and after a strong repulsion force is added. This sample is with the strongest repulsion magnitude we use, $k=20$. A wide peak two small particles’ radii away (0.2 units) before repulsion becomes a tall, narrow peak after repulsion. The same thing, to a lesser degree, is seen at 0.4 units. The particle density function is normalised so that $g(r)\rightarrow 1$ as $r \rightarrow \infty$. The distances are nondimensionalised on $a_\mathrm {large}$

It is convenient to quantify the structure of the suspension by looking at the particle density function (PDF, or pair distribution function, or percolation function) [9, 13]. This function, commonly notated *g*(*r*), counts the number of particles a distance *r* from a reference particle at a given time, averaged over a set of reference particles. Figure 6 shows, on the left, the PDF for a randomly generated fully 3D field of small particles. No particles can be closer than the depth of two particles’ radii (otherwise, they would overlap), and we see a peak in the number of particles at this closest value of 0.2, with a fairly wide tail. There is a smaller shallow peak at the next band, 0.4. The right-hand figure shows the PDF after 25 frames of strong repulsion: the first peak is now much taller and narrower, as the bands are now better defined, but otherwise the repulsion does not have an effect on the structure of the suspension. Weaker repulsion has a less-pronounced effect.

In our simulations, the repulsion force is the only non-hydrodynamic force applied to the small particles in the system, which are otherwise passive. We find this to be sufficient to reproduce some of the features seen in experiment. However, other pairwise forces can be added, which can have interesting consequences, most notably the role of frictional contact forces in shear thickening [12], not discussed here.

## Setting the size of the box

Our large particle sediments through a box of smaller particles. This box is not periodic, as it is not clear how the suspension bulk would be affected by the unwanted reproductions of the heavy sphere above and below the box. Outside this box, we have simply quiescent fluid. In order to balance the requirement for a large box size with the availability of computational resources, we set the box size so that as the large sphere sediments, we see no vertical motion at the edges of the box.

**Fig. 7:**
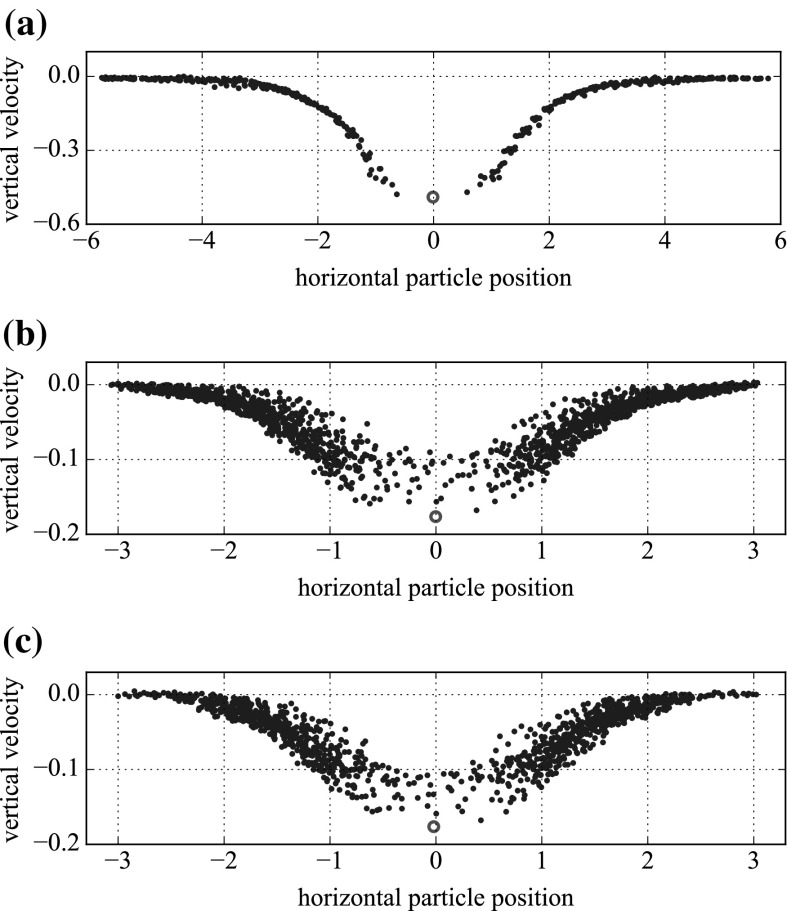
Vertical velocities of small particles at the same depth as the sedimenting large particle (with a centre-to-centre height difference of up to $a_\mathrm {large}$) at a representative depth. The velocity of the large particle is marked on $({\circ })$ for reference. The horizontal measurements are nondimensionalised on $a_\mathrm {large}$. **a** through a monolayer suspension. **b** through a fully 3D suspension, without walls at the sides. **c** through a fully 3D suspension, with walls at the sides

We confirm this in Fig. 7. Here, we have plotted the vertical velocities of the small particles at the same approximate depth as the sedimenting particle through a monolayer and through fully 3D suspensions, with and without walls at the sides. The velocities were captured when the sedimenting particle was approximately halfway through the sample, but the plot is similar at all depths, repulsion strengths and shear rates. At the edges of the box, the vertical velocities of the particles are almost zero, as preferred. This effect is only slightly emphasised in the presence of walls. In a three-dimensional (3D) simulation, a box width of $6a_\mathrm {large}$, as in subfigures (b) and (c), requires around 10,000 small particles.

## Sedimentation in unsheared suspension

To quantify the effect of an applied shear, we first perform some simulations of the large ball falling in an unsheared suspension.

### Monolayer unsheared suspension

**Fig. 8:**
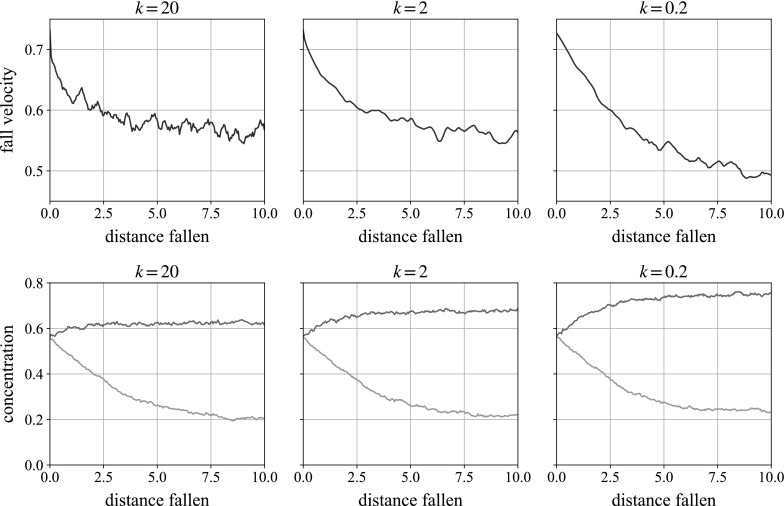
*Top* A large ball sedimenting through a unsheared monolayer suspension, at different repulsion strengths, slowing down as it falls. In contrast to Fig. 4, here we have given the suspension some time to equilibrate after applying the repulsion force, before activating gravity on the large ball. *Bottom* The concentrations of small particles behind (*lower line*) and ahead of (*upper line*) the falling ball as it falls. The concentration is measured in a box the width of the particle and of dimensionless height 8, centred on the falling ball

As we shall later examine a sphere sedimenting in a sheared monolayer, a reasonable approximation when gravity acts in the plane of shear (i.e., when gravity acts in the negative *x*- and *y*-directions in Table 2), we start by letting a sphere fall through a monolayer of randomly assembled smaller beads. We plot in Fig. 8 the transient fall speed of the large sphere (normalised by its Stokes velocity in pure solvent) as it sediments through this suspension. We find that for three well-differentiated repulsion strengths, the falling ball decelerates before achieving a mean falling velocity which has only a weak dependence on the repulsion magnitude, *k*. In contrast to Fig. 4, here we have given the suspension some time to equilibrate after applying the repulsion force, before activating gravity on the large ball.

The deceleration is caused by the build-up of concentration beneath the ball, which is also quantified in Fig. 8. With the local area concentration starting at $c\approx 0.55$, we see that below the particle (upper line), the concentration increases until finding an equilibrium level: from $c \approx 0.63$ at the highest repulsion to $c \approx 0.72$ at the lowest. In the lowest repulsion sample, the area concentration is still comfortably below the maximum circle packing fraction of 0.91 with hexagonal packing, but is approximately the square packing fraction of $\pi /4 \approx 0.79$. The concentration behind (lower line) initially decreases, as the falling ball creates a wake, before increasing again as the wake enters the more concentrated zone created by the falling ball. The wake is unexpected normally in reversible Stokes flow, but occurs due to the repulsive force, which is inherently irreversible.

### Fully 3D unsheared suspension

**Fig. 9:**
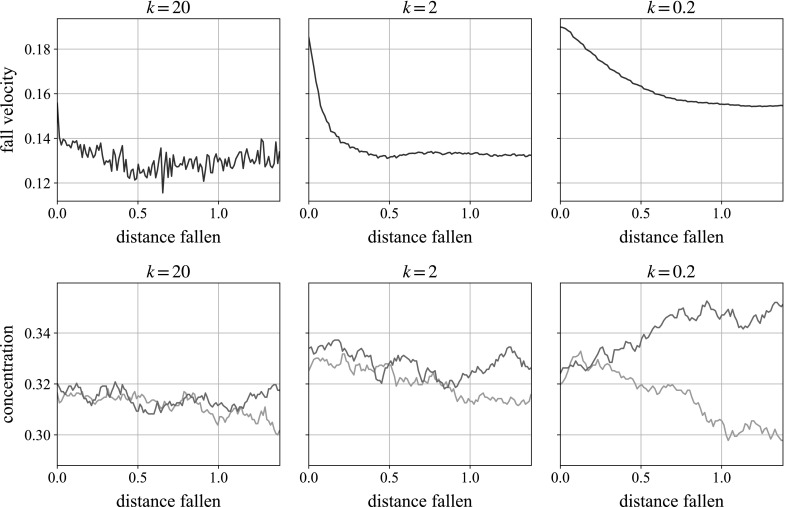
*Top* A large ball sedimenting through a 3D unsheared suspension, at different repulsion strengths, slowing down as it falls. Weaker repulsions lead to faster falling speeds. *Bottom* The concentrations of small particles behind (lighter, predominantly *lower line*) and ahead of (darker, predominantly *upper line*) the falling ball over time. The concentration is measured in a cube, by the width and depth of the particle, with dimensionless height 3, centred on the falling ball

We perform the same test on a cube of small particles containing the sedimenting sphere. In contrast to the monolayer simulation, the falling velocity and concentration in Fig. 9 stabilise much more quickly. We see a quick deceleration for all three repulsion strengths before reaching a fairly constant velocity. The mean falling velocity is shown to have a stronger dependence on the repulsion strength, *k*, than in the monolayer, and in fact suggests the opposite dependence: weaker strengths lead to faster mean velocities. In full 3D, the number of neighbouring particles—the ‘kissing number’—is much higher than in a monolayer, and the slower falling speeds with a higher repulsion force can be explained by higher (upwards) repulsion from the large number of smaller particles beneath the falling ball.

The concentration build-up beneath the ball is also shown in the same figure. As expected, behind the ball (*lower line*), we see a reduction from the starting volume concentration of $\phi \approx 0.33$ to $\phi \approx 0.30$. Unlike in the monolayer simulations, however, the concentration in front of the ball (*upper line*) shows very little change. This can again be rationalised by the larger number of neighbours beneath the ball in full 3D giving stronger resistance to compression, as well as the extra available space for them to move into in full 3D.

## Oscillatory preshear

In the experiments from Blanc et al. [8], it was found that a large ball, sedimenting in a oscillatory sheared suspension, can sometimes slow so much as to travel upwards over the course of a shear cycle. Given that the hypothesised mechanism is in the structure created by the shear, we set out here to investigate the form of this structure in our simulations. In line with the experiments, we preshear the suspension with two oscillations. This also allows us to observe the effect this shear has on a neutrally buoyant large sphere.

We place the large sphere towards the top of the cube of smaller particles, ready for release, but for now make it neutrally buoyant. We have conducted tests where walls of particles have been added along the neutral planes, but we have found that this makes only a small difference to the behaviour of the large sphere.

### Effect of initial position relative to shear

**Table 3:**
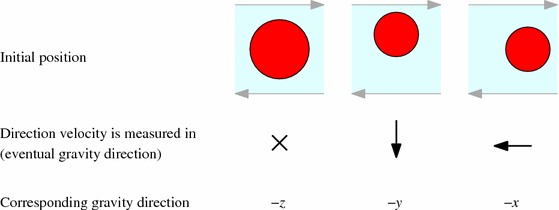
Summary of the three initial positions relative to the shear that we perform preshear, neutrally buoyant simulations on

We perform one 3D preshear simulation for each of the three gravity directions shown in Table 3. In each, the initial position of the large sphere is set to be towards the ‘top’ of the cube of smaller particles, according to what will be the fall direction during sedimentation. The large sphere, ready for release, is for now made neutrally buoyant. The velocity of the sphere is then measured in what will be the gravity direction, so that we may compare this shear-induced motion to the motion under sedimentation.

**Fig. 10:**
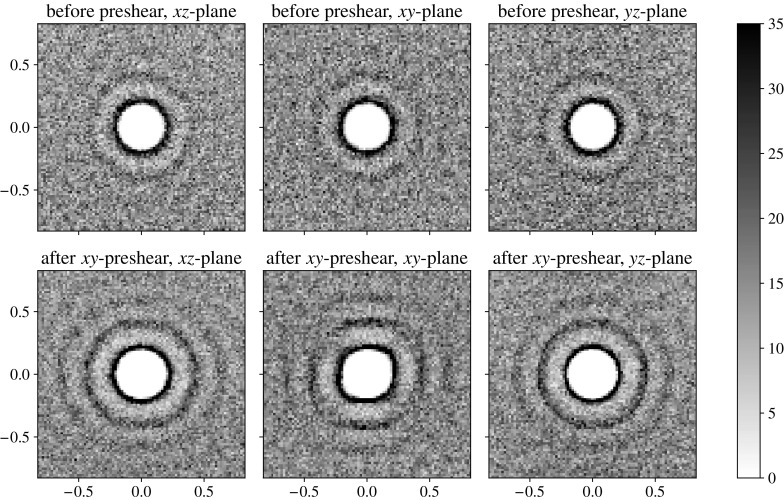
Particle density function plots in three planes before and after two oscillations of *xy*-preshear in a fully 3D simulation. We see that preshear emphasises the circular structure in the neutral planes (*xz* and *yz*, *left* and *right figures*), but again forms square packing in the shear plane (*xy*, *central figure*). The slices in the plane have depth of two small particles’ radii. The repulsion and frequency choice is $k = 20$, $f = 0.1$

Applying two cycles of shear in each simulation, we find that the oscillatory preshear creates structure in the suspension bulk. Figure 10 shows the particle density functions of slices in each plane before and after an oscillatory shear (which, recall, is always in the *xy*-plane). We see that the preshear slightly emphasises the circular structure near the centre of the plots in the neutral planes, whereas in the shear plane, we see distinctive alignment of particles parallel and perpendicular to the shear direction: square packing.

The observation of square packing agrees with the hypothesis in Blanc et al. [7]: that particles align in the vertical direction under oscillatory shear. However, as we shall see, in our simulations, this does not lead to faster sedimentation.

**Fig. 11:**
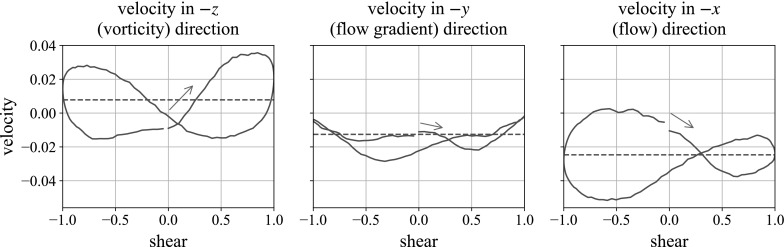
Velocity plots of the neutrally buoyant large sphere in oscillatory shear in a fully 3D suspension. Here we see the velocities in the gravity direction for each of the three simulations in Table 2. All three plots are for the moderate parameters $f=1$, $k=2$. The *horizontal dashed line* shows the mean velocity

We now look at the velocity of the large, neutrally buoyant sphere, in a cube of smaller particles, in our three gravity directions. The variations of the velocity as a function of phase during a shear cycle—from now on, ‘velocity plots’—are shown in Fig. 11. In each, the velocity of the large sphere is plotted against the system shear for the second oscillation, and the horizontal dashed line marks the mean velocity. The oscillation begins with motion to the right, and we plot *falling* velocity as positive. We find that in all cases, the mean velocity over a shear cycle is negative. That is to say, the ball, when neutrally buoyant, travels upwards over the course of the shear. This is purely an effect of our asymmetric geometry: the large ball is placed near the top of the region occupied by particles, so it is driven upwards by the repulsion from the larger number of particles below it than above it. Furthermore, in all cases, the ball translates vertically, mostly in a figure-of-eight shape, although in the flow gradient direction, where the variation in velocity is smaller, this is less clear. Interestingly, we observe that velocity plot in the vorticity direction traverses a figure of eight in the opposite direction to the velocity in the flow direction. We will discuss this observation further in Sect. 8. We also find that the velocity changes are of similar magnitude for sedimentation in the flow and vorticity directions, but are opposite in sign.

### Effect of shear frequency and repulsion

**Fig. 12:**
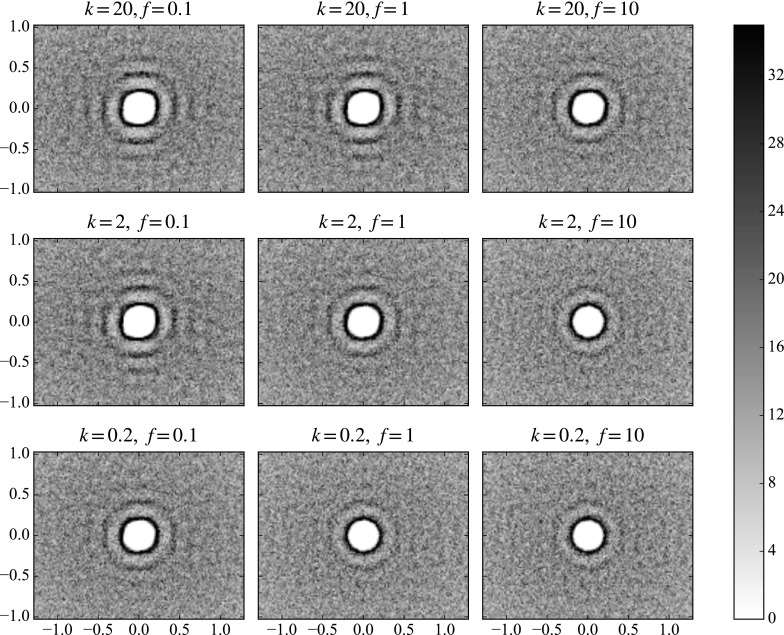
Particle density function plots for oscillatory preshear after two oscillations in a fully 3D simulation. Plots with low values of *k* / *f* (*bottom right*) show little difference to their initial state (see Fig. 6), whereas higher values of *k* / *f* (*top left*) display distinctive square packing. The slices in the plane have depth of 2 small particle radii

**Fig. 13:**
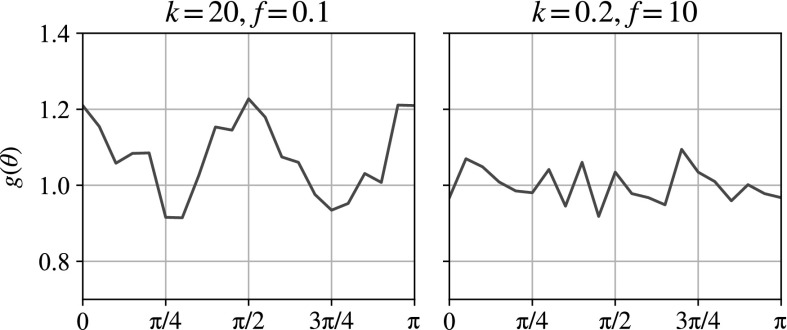
Two particle density function plots as a function of angle at a constant radius band corresponding to the second dark band in Fig. 12 ($0.39a_\text {small} \lesssim r \lesssim 0.45a_\text {small}$) after two oscillations of preshear in a fully 3D simulation. By the symmetry expected, we only plot from $\theta =0$ (the positive *x*-axis in the PDF plots in Fig. 12) to $\theta =\pi$. The low-frequency, high-repulsion case on the left shows peaks at 0, $\pi /2$ and $\pi$, corresponding to the observed square packing. The particle density function is normalised so that the mean of the right-hand plot is 1. Particles are counted into 40 equal-sized bins of $\theta$, and the average of three simulations is shown

We examine the importance of the shear frequency and repulsion strength on a fully 3D sample. The particle density function plots in the shear plane are shown in Fig. 12. Here we see nine parameter combinations: simulations with different repulsion strengths, *k*, and different oscillation frequencies, *f*. Note that since we nondimensionalise the frequencies in terms of the weight of the falling ball in Eq. (4), there is ambiguity when the ball is neutrally buoyant. Although we show all nine parameter combinations for comparison with the later sedimentation phase, we note that here there is only one independent parameter, *k* / *f*, which is demonstrable in the diagonal symmetry in the plots.

We see that in the shear plane, the imposed oscillatory shear creates a square-packing structure in the suspension bulk. At low repulsions and high frequencies, there is little difference from the circular structures seen prior to shearing (see Fig. 6), but the effect is clearer for the increasing repulsion strength and the decreasing frequency. We can quantify the degree of square packing in the particle density function plots as a function of angle round the second dark band, $g(\theta )$. Figure 13 shows two extreme cases. The right-hand plot—the high-frequency, low-repulsion case—shows a mostly constant concentration of particles around the band, but the left-hand plot—the low-frequency, high-repulsion case—shows peaks at $\theta = 0$, $\pi /2$ and $\pi$, corresponding to the observed square packing.

## Sedimentation in oscillatory shear

We now apply a weight force to the large sphere while continuing to shear the suspension.

### Effect of gravity direction relative to shear

**Fig. 14:**
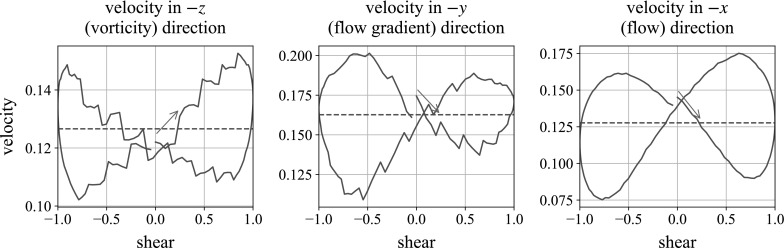
Velocity plot of the sedimenting heavy sphere in oscillatory shear, corresponding to the geometries in Table 2. All three plots are for the moderate parameters: $f=1$, $k=2$. The *horizontal dashed line* shows the mean velocity over the shear cycle. The stepping in the first two plots is an artefact of the method

The velocity plot of the falling sphere in the system, sedimenting in each of the three directions, is shown in Fig. 14. In all three gravity directions, the fall velocity of the ball oscillates over a shear cycle. Note that sedimentation in the flow gradient and flow directions leads to figures of eight which are traversed in the opposite sense to that in which it is traversed when the ball sediments in the vorticity direction: in the first two cases, the ball slows down during the central half of the shear cycle (between $-0.5$ and 0.5), whereas in the latter case, it speeds up. We describe a mechanism for this behaviour in Sect. 8. The third figure in Fig. 14 therefore reproduces the figure-of-eight shape observed in experiment, but traversed in the opposite direction. The ‘steps’ in the first two figures are a sensitivity to a detail of the Stokesian Dynamics method—specifically, how often we calculate the long-range mobility matrix. Increasing how often we calculate this gives smoother plots, as in Fig. 11, but comes with the considerable time expense of simulation.

The velocity plots appear to be a sum of the unsheared falling speed, Fig. 9, and the oscillatory behaviour from the shear, Fig. 11. We do not observe significant changes in the mean velocity from the unsheared case.

### Effect of frequency and repulsion strength

We now look at the effect of changing the frequency and repulsion strength. Some elucidation of the effects at work here is provided by examining the repulsive energy in the system over time. This may be calculated from the magnitudes of the repulsive forces in the system, for the exponential force we are using (Eq. (5)): the repulsive energy, *E*, for a pair of particles *i* and *j* with surface separation $h_{ij}$ is the work done against the repulsive force in bringing them to that position, starting from far apart:

6 $$\begin{aligned} E(i,j) = \int _{h_{ij}}^\infty {{\varvec{F}}} \cdot \mathrm {d} \varvec{r} = \frac{1}{\tau }| {{\varvec{F}}}(h_{ij}) |. \end{aligned}$$

This is then summed over all pairs of particles in the system.

**Fig. 15:**
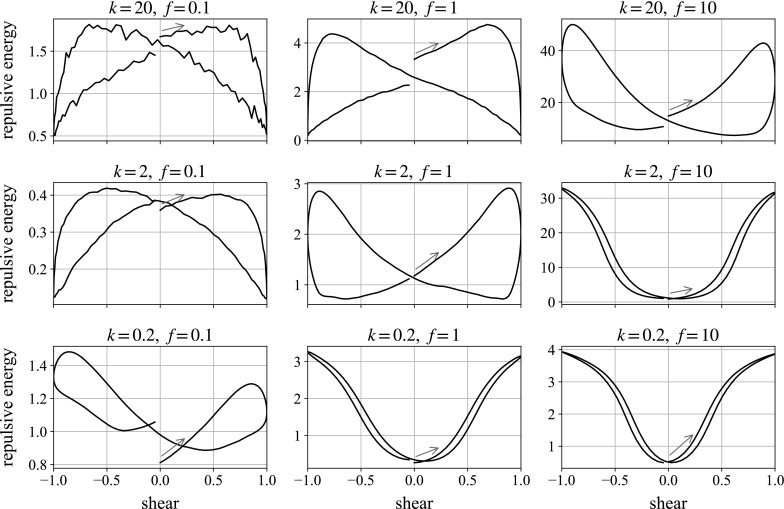
Monolayer simulations of a ball falling in the flow-gradient direction. We plot the total energy in the system for the second shear cycle against state of shear. In each plot, the oscillation begins with motion to the *right*, following the *blue arrow*. (Color figure online)

Figure 15 measures the repulsive energy in a monolayer system over the second oscillation of shear as the ball sediments in the velocity gradient direction (see Table 2) for different parameter values. We see broadly three regimes. The first, in the bottom-right of the figure, is a U-shape: the high-frequency, low-repulsion regime. Starting from the centre of the plot and moving to the right, the repulsion energy increases as the system is sheared and compressed, and then reverses mostly symmetrically as the system is unsheared and uncompressed. In this limit, the oscillation is too fast for the repulsion to act: the hydrodynamic forces associated with the shear dominate over the weight and repulsion forces, essentially making the particles passive. At high frequencies and low repulsions, the energy plot is therefore reversible. We begin to see hysteresis above and to the left of this plot, as decreasing the frequency gives the repulsion time to act, and increasing the repulsion forces no longer leaves the shear forces dominant.

These hysteresis loops are emphasised in the central plot in the figure—with intermediate parameter values—where we have a figure of eight. Again starting from the centre of the plot and moving rightwards, the system is compressed as it is sheared, building up structure and repulsion energy. At the end of the shear, though, the repulsion force has time to act, whereas it did not in the previous regime. The particles repel each other, the structure is destroyed, and the system relaxes. Then, as the system is sheared in the opposite direction, the particles are compressed again and the structure and energy increases. This alternating between construction and destruction generates the figure of eight.

Finally, the bottom-left plot of the same figure—low repulsion, low frequency—shows ascending loops. This regime is just a slower version of the aforementioned one: the system has time to relax when the shear direction changes, but as shears take longer with low frequencies, the concentration builds up more beneath the falling ball during the shear cycle and thus the system repulsion energy increases over time.

**Fig. 16:**
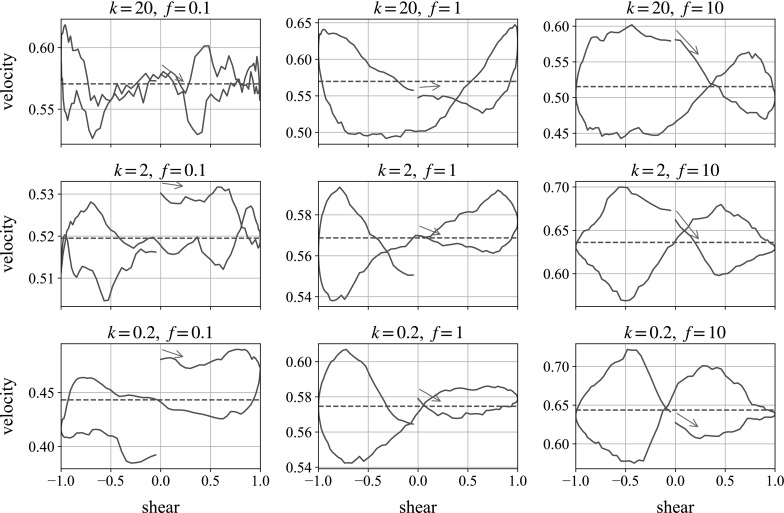
Monolayer simulations of a ball falling in the flow-gradient direction. We plot the instantaneous fall speed against state of shear. In each plot, the oscillation begins with motion to the *right*, following the *blue arrow*. The *dotted horizontal line* marks the mean falling speed over the shear. (Color figure online)

These different regimes loosely match with the velocity plots shown in Fig. 16, measured over the same single oscillation of the shear flow for the same nine different parameter combinations. Again, the oscillation begins with motion to the right, and we plot falling velocity as positive.

Starting from the bottom-left, the low-repulsion, low-frequency case, we see the velocity decrease over time as the concentration builds up beneath the ball, agreeing with the loops in the repulsive energy plot. In the central and right-hand plots we see figures of eight, as the particle slows down under the increased repulsion from the structured, compressed suspension. As the system relaxes and the structure is lost—the small particles ‘space out’ under repulsion—the large particle is able to fall with less hindrance, and its speed increases until the reverse shear increases the local concentration and repulsion forces again.

Looking from left to right across the figure, we can see that increasing the frequency increases the velocity difference over the shear: speeding up the oscillation by a factor of 10 leads to an approximate doubling of the percentage velocity gain. This suggests that increasing the frequency of oscillation further, or reducing the weight of the falling ball, should lead to greater velocity differences, allowing the particle to rise over parts of the shear. This would mimic the behaviour seen experimentally. These results are all for sedimentation in the velocity gradient direction through a monolayer. It is not until we shear a fully 3D cell, simulating the experiments, that we see the ball travel upwards.

**Fig. 17:**
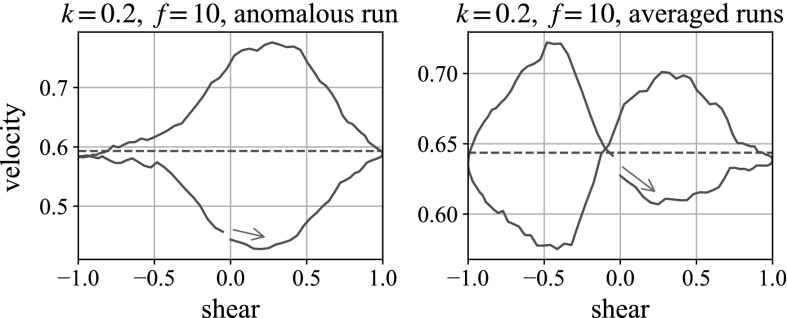
Individual simulation for parameters $k=2$, $f=10$, compared to a plot averaged over multiple runs

The plots shown are averages of two simulations with different initial suspension distributions. It is worth noting that although on average, the plots give figures of eight, individual runs can be sensitive to initial configurations, producing anomalous plots. We understand that this is also an occasional feature of the experiments. We find that the bottom-right parameters in Fig. 16 (low repulsion and high frequency) is the most likely to produce such results, and an example is compared with the average in Fig. 17.

Here, we see behaviour that appears counterintuitive: when the shear is moving to the right the ball accelerates, but when the shear is moving to the left it decelerates, which would appear to break the intrinsic symmetries of the system. However, with both repulsive forces and the weight of the ball being very small in this limit compared with the hydrodynamic forces associated with the external shear flow, all the particles are essentially passive, and simply move with the shear flow, hindered by each other’s presence in a purely hydrodynamic way. The falling ball is pushed primarily horizontally, with small perturbations in the vertical direction. One of these is caused by the presence of the small particles, which happen (for this simulation) to be arranged in such a way that the perturbation is upwards when the motion is to the left and (by reversibility) downwards when the motion is to the right. The other is caused by the particle’s weight, which always drives it downwards. Because we have made velocities dimensionless using the Stokes velocity of the large sphere in the absence of small particles, these velocities both appear order 1 in our plots.

**Fig. 18:**
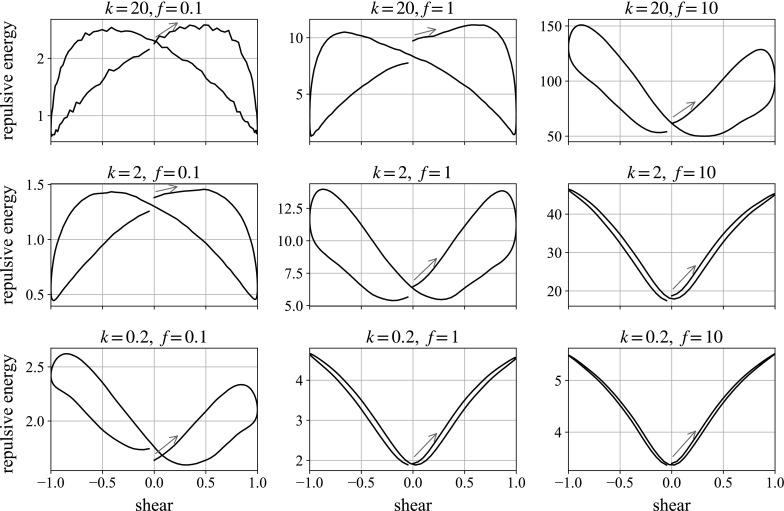
Three-dimensional simulations of a ball falling in the vorticity direction. We plot the total energy in the system against state of shear for the second oscillation. In each plot, the oscillation begins with motion to the right, following the *blue arrow*. (Color figure online)

**Fig. 19:**
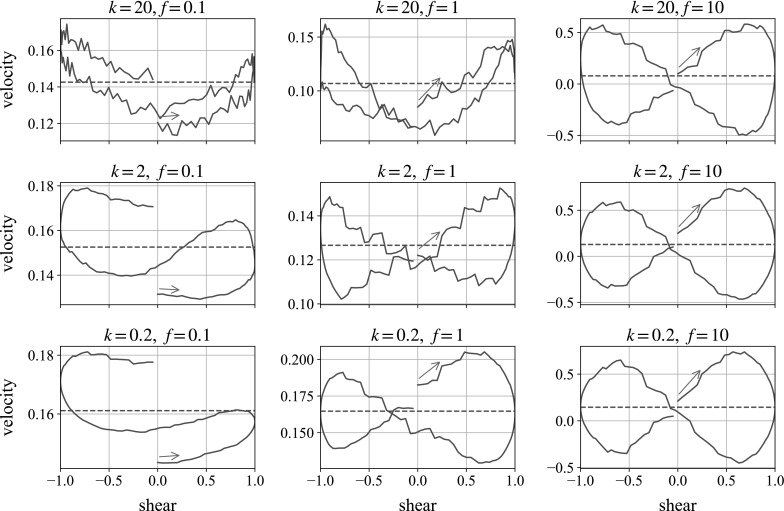
Three-dimensional simulations of a ball falling in the vorticity direction. We plot the instantaneous fall speed against state of shear for the second oscillation. In each plot, the oscillation begins with motion to the *right*, following the *blue arrow*. The *dashed horizontal line* marks the mean falling speed over the shear. (Color figure online)

Figures 18 and 19 show the repulsive energy and velocity plots for the falling sphere in a cube of smaller particles, undergoing oscillatory shear. The repulsive energy is of a similar magnitude to in the monolayer case, but the loops retain a stronger U-shape in the moderate parameter values. The velocity plots produce more consistent figures of eight in the central and right-hand column, allowing for the appearance of steps which are an artefact of the method. This is not surprising, given that the increased number of neighbours in fully 3D suspensions offers more consistent resistance to the movement of the large sphere. As mentioned when looking at preshear, the velocity plots are traversed in the opposite sense to which it is traversed in the monolayer simulations. In this geometry, the build-up of structure corresponds to the ball speeding up, which is lost when the structure is destroyed. The mechanism is discussed fully in Sect. 8. The left-hand plots are mostly unaffected by the slow shear, showing the velocity slowly increasing during the shear: this simply reflects the large particle getting closer to the bottom of the simulation box over the shear period, thus having fewer particles in its way.

Some observations are the same as in the monolayer: increasing the frequency by a factor of 10 leads to a similar proportional increase in the velocity difference over the shear cycle. In our high frequency cases, this leads to the ball traversing upwards over the course of the cycle: the same behaviour seen in the experiments, although with the plot again traversed in the opposite direction. Changing the repulsion strength does not seem to lead to significant changes in the plots.

### Effect of concentration and amplitude

**Fig. 20:**
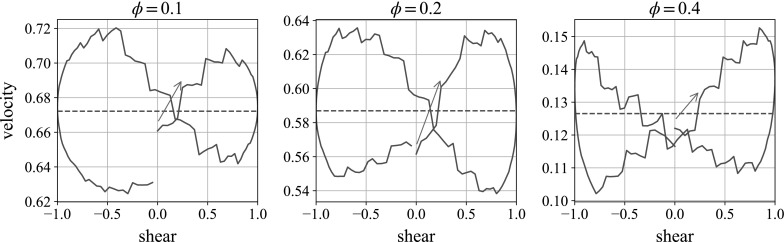
Velocity plot of the sedimenting heavy sphere in oscillatory shear. Here we see the velocities in the gravity direction. The volume concentrations are $\phi =0.1$, 0.2 and 0.4 (used in the rest of the study). All three plots are for the moderate parameters $f=1$, $k=2$. The *horizontal dashed line* shows the mean velocity over the shear cycle

The concentration of the suspension is varied in Fig. 20 to $\phi =0.1$ and 0.2, as well as the existing $\phi =0.4$ which we use for the rest of the study. Here the ball is falling in the vorticity, or negative *z*-, direction (see Table 2), with the moderate frequency and repulsion parameters $f=1$, $k=2$. We see that, as expected, decreasing the concentration increases the mean fall velocity. However, it does not appear to dramatically change the variation in velocity over the shear, which remains at approximately $\pm 0.05$.

**Fig. 21:**
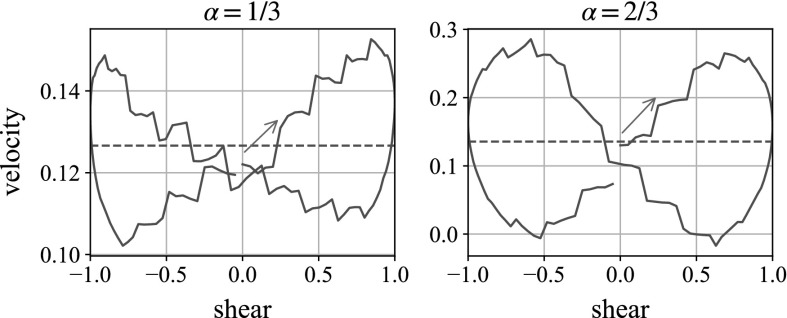
Velocity plot of the sedimenting heavy sphere in oscillatory shear. Here we see the velocities in the gravity direction. The amplitudes of the oscillatory oscillation are 1 / 3 (as in the rest of the study) and 2 / 3. Both plots are for the moderate parameters $f=1$, $k=2$. The *horizontal dashed line* shows the mean velocity over the shear cycle

**Fig. 22:**
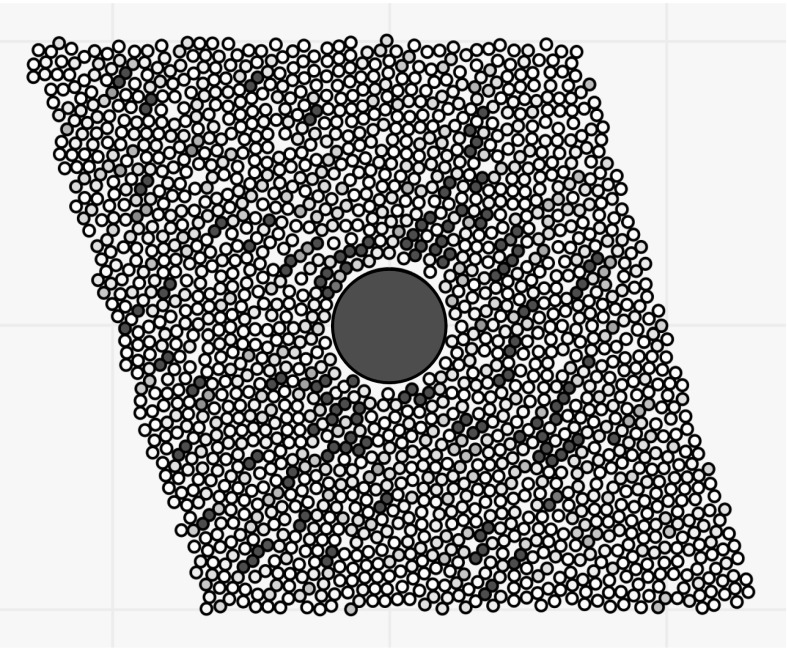
Chains of particles forming parallel to the compression axis. A monolayer of particles in the *xy* plane is shown midway through the shear cycle, in the absence of gravity. Small particles experiencing large-magnitude forces are coloured in *red*, indicating the chains of repulsion. The parameters used are $k=2$, $f=1$, in addition to those from Table 1. (Color figure online)

The amplitude of the shear is varied for the same moderate parameters in Fig. 21. We see that the mean velocity of the falling sphere over the shear cycle is mostly unchanged from the unsheared suspension, but the variation in velocity across the shear is increased by increasing the shear amplitude proportionally. This makes sense: increasing the amplitude increases the energy put into the system.

## Mechanism behind velocity plots

For a sphere sedimenting in the flow gradient and flow (negative *x*- and *y*-) directions, we see the ball slowing down during the central half of the shear cycle and speeding up at the extremes. For a sphere sedimenting in the vorticity (negative *z*-) direction, we see the ball speeding up during the central half of the shear cycle and slowing down at the extremes.

It is tempting to argue on energy lines, that the slowing falling velocity, and hence the height gain in Blanc et al. [8], must come from a release of the elastic energy associated with the repulsive forces. However, as we see in the central case in Figs. 15 and 16, this is not the case. Here, the maximal rate of release of elastic energy (i.e. the maximums of the plots, at shears of $\sim {\pm }0.9$) actually coincides with downwards acceleration of the large sphere, where the relaxing system allows the ball to fall with less hindrance. There is no paradox here: the imposed shear flow is an energy source which adapts instantaneously to the energy being dissipated by viscous forces.

Instead, we break the oscillatory shear, and hence the velocity plot, into two periods: a structure-creating period, as the system is sheared; and a destruction period, as the shear direction changes, when the system is allowed to relax. Whether this causes the falling sphere to accelerate or decelerate depends on the direction of sedimentation relative to the shear, and to a lesser extent, the amplitude of shear.

**Fig. 23:**
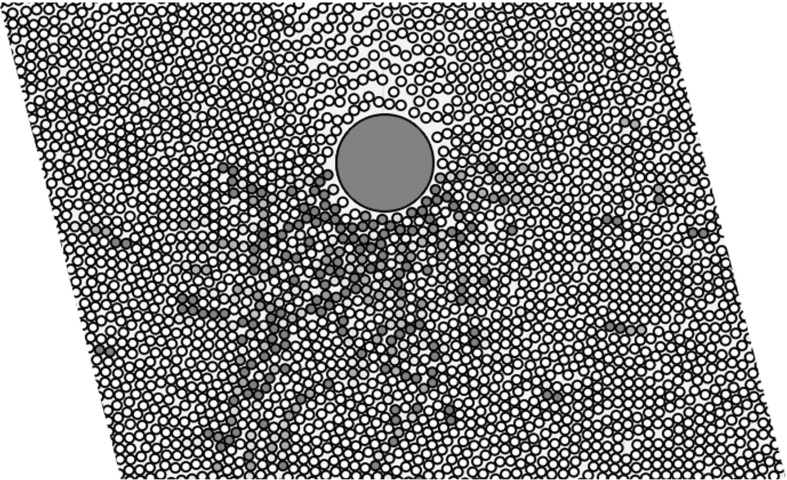
Chains of particles forming beneath and to the side of the ball, adding upward force. A monolayer of particles in the *xy* plane is shown midway through the shear cycle, after the ball has fallen under gravity some distance. Small particles experiencing large-magnitude forces are coloured in *red*, indicating the chains of repulsion. The parameters used are $k=2$, $f=1$, in addition to those from Table 1. (Color figure online)

We have already seen in Sect. 6.1 that as the system is sheared, it forms a square-packing structure in the shear plane. In the same plane, we also see the formation of ‘chains of repulsion’. Figure 22 shows this effect in the plane of preshear: small particles lying parallel to the compression axis experience high repulsion forces (marked in red). Since in our systems, the ball is placed near the top of the system, there are more small particles beneath the ball than above it, giving us longer, stronger chains. The net effect of these chains is to push the ball to areas of less concentration (which lie above it).

For sedimentation in the shear plane, we see, as in Fig. 23, that as the particle falls, it builds up concentration beneath it. Simultaneously, shearing the suspension causes these chains of repulsion to form. As chains form in more concentrated areas, there are more chains in front of the falling particle than behind it, and there are more along the compression axis than in the uncompressed axis. The heavy ball is pushed by these chains of repulsion towards less concentrated areas (which again lie above it), which is why it slows down—and, given long enough chains of repulsion, can make it move upwards.

**Fig. 24:**
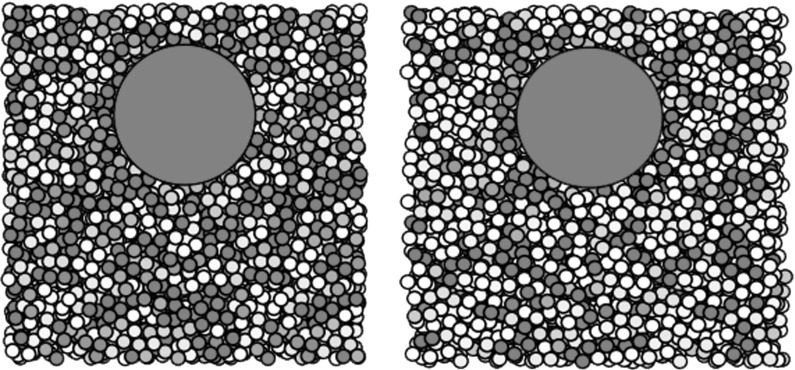
A vertical slice (in the *xz* plane) through the centre of a cube of small particles (like half an avocado with the stone left in), with a neutrally buoyant large particle, midway through a shear cycle. Small particles experiencing large-magnitude forces are coloured in *red*. The parameters used are $k=2$, $f=1$, in addition to those from Table 1. *Left* Before shear. *Right* During shear, when vertical chains form. (Color figure online)

When the ball sediments in the vorticity direction, we see a figure of eight in the velocity plots of a similar magnitude but traversed in the opposite direction. Again, chains of repulsion are formed, but these have a reduced effect, as they lie predominantly in the shear plane. Instead, the square-packing structure is responsible for the acceleration over the shear. As seen in Fig. 24, the shear causes vertical chains to form parallel to the gravity direction. In sedimentation, this makes it easier for the ball to fall through, relative to the unsheared suspension. When the shear direction changes, the structure is destroyed and the ball slows down again, in places being pushed upwards by the repelling particles. In preshear, the net force on the large sphere from the small particles is upwards (seen by the mean upwards velocity in Fig. 11), provided by the larger number of particles beneath the large sphere. Over the shear, structure forms which reduces this net effect, causing the ball to slow down over shear. As the shear direction changes, the structure is again destroyed and the net upwards force accelerates the ball upwards.

We see in Fig. 21 that the velocity plots are dependent on the amplitude of shear. Increasing the amplitude of oscillation increases the energy put into the system, which explains the increases velocity differences at higher amplitudes.

Furthermore, that in Fig. 20 we saw little difference to the velocity difference for different concentrations is initially surprising, given the mechanism described above depends on structure, which should be enhanced by concentration. But with reduced concentration, the mean velocity is higher (so proportionally the velocity difference is reduced), so the falling ball meets more particles over a shear cycle, producing a similar effect to an increased concentration.

## Conclusions

The experiments by Blanc et al. [7] posed multiple questions about the behaviour of the microstructure in an oscillatory sheared suspension. Our Stokesian Dynamics simulations offer explanations for some of their observations. In particular, we are able to produce figure-of-eight velocity plots, although traversed in the opposite sense to that it is traversed in the shear geometry most closely matching the experiments. We also have a feasible mechanism—repulsion chains in the plane of shear, breakdown of vertical chains perpendicular to the plane of shear—for which the upward motion in the shear, observed in the experiments, is possible. We have also shown that the behaviour over a shear cycle is strongly affected by the imposed frequency and repulsion strength, giving different regimes for the velocity plots. That we have not seen the increased fall velocity in the heavy sphere suggests that another mechanism is responsible for this, or at least that it requires more than just repulsion between particles. The distances travelled by the ball in experiments are up to 20 times larger than the box size in our simulations, and the number of shear cycles experienced by the falling ball far higher, so it is feasible that observing this increased fall velocity requires additional particles with higher build-ups of structure.
